# Medical Society of the County of Erie

**Published:** 1907-12

**Authors:** Franklin C. Gram


### SOCIETY PROCEEDINGS.

---

### Medical Society of the County of Erie

Reported by FRANKLIN C. GRAM, M. D., Secretary

THE annual meeting of this society was called to order by first vice-president Dr. Edward Clark (the president being absent from the city), on Monday, October 14, 1907, at 4 p.m.

The secretary read the minutes of the last meeting, and also those of the council which were duly approved.

The chair asked the pleasure of the Society regarding the manner of the annual election of officers to be held, stating that it had been customary to appoint a nominating committee, although the by-laws provided for election by ballot; whereupon Dr. Bonnar moved that the society proceed to elect officers by direct nominations. Carried.

Dr. Rochester nominated Dr. Charles A. Wall for president. Dr. Dunham nominated Dr. Edward Clark for president. Nominations were then declared closed, and the chair appointed Drs. Lytle and Bennett as tellers.

The tellers announced 32 votes cast, of which Dr. Clark received 17 and Dr. Wall 15. Dr. Clark was, therefore, declared duly elected president of the society for the year 1908.

It was moved by Dr. Fronczak that the two vice-presidents be chosen on one ballot, the one receiving the highest number of votes to be declared first vice-president, and the next highest, second vice-president. Carried.

Dr. Grant nominated Dr. Charles A. Wall; Dr. D. C. Greene nominated Dr. James S. Smith; Dr. Bennett nominated Dr. Grover W. Wende. Nominations were then declared closed. The total number of ballots cast was 31, of which Dr. Wall received 26 votes, Dr. Grover W. Wende received 24 votes, and Dr. James S. Smith 12 votes. Dr. Charles A. Wall was then declared duly elected first vice-president, and Dr. Grover W. Wende, second vice-president.

Dr. Eli H. Long nominated Dr. F. C. Gram as secretary, this nomination being seconded by Dr. W. D. Greene. Dr. Rochester nominated Dr. William Irving Thornton as secretary. Nominations were declared closed. Dr. Thornton withdrew his name, and Dr. Benedict moved that the chair cast the ballot of the society for Dr. F. C. Gram as secretary.

Objection being made by Dr. Grosvenor, the chair ruled that each member must prepare his ballot. The tellers announced 31 votes cast, of which Dr. Gram received 19, Dr. Rochester 1, Dr. Hartwig 2, Dr. O'Gorman 1, Dr. Blaauw 1, Dr. Thornton 1, Dr. Bennett 1, Dr. Grover W. Wende 1, with 4 blanks. The chair thereupon declared Dr. Gram duly elected as secretary.

Dr. Dewitt C. Greene, the present treasurer of the society, stated that he no longer desired to continue in the office and nominated Dr. Albert T. Lytle as treasurer. No other nominations being made, the chair declared the nominations closed. The chair appointed Dr. Dunham as teller in place of Dr. Lytle. Twenty-nine votes were cast for treasurer, of which Dr. Lytle received 21, Dr. Rochester 3, Dr. Gram 1, Dr. Gray 1, Dr. Greene 2, and blank 1. The chair then declared Dr. Lytle duly elected as treasurer.

Dr. Eli H. Long moved that the election of remaining officers and committees be deferred until after the reading of a paper by Dr. John P. Sawyer of Cleveland, Ohio, who was present as the guest of the society. Dr. Sawyer then read an interesting and instructive paper on diabetes. The paper received the close attention of all the members present, and at its close it was discussed by Dr. Rochester, who afterward moved a vote of thanks to Dr. Sawyer for his admirable address.

Further nominations and elections were then resumed. Dr. O'Gorman and Dr. Dunham were appointed tellers, and the result of subsequent elections was as follows: Board of Censors, Dr. Henry R. Hopkins, chairman; members, Drs. DeLancey Rochester, Francis E. Fronczak, Walter D. Greene and John H. Grant.

Dr. Bennet declined to serve further as chairman of the committee on legislation, and Dr. A. A. Hubbell was elected as chairman of such committee. Dr. Ernest Wende was elected chairman of the committee on public health. Dr. T. H. McKee was elected as chairman of the committee on membership.

Delegates to the Medical Society of the State of New York for two years were elected as follows: Drs. George L. Brown, Arthur W. Hurd, William H. Thornton and Francis M. O'Gorman.

Delegates to the Eighth District Branch of the Medical Society of the State of New York, for two years, were elected as follows: Drs. Walter D. Greene, J. F. Rice, Chas. A. Wall, Francis E. Fronczak and S. A. Dunham.

Dr. A. T. Lytle of the committee on membership reported favorably upon the following candidates: Dr. Arthur Porter Squire, 277 Franklin Street, Buffalo; Dr. W. Warren Britt, 17 Kohler Street, Tonawanda; Dr. John A. Ragone, 254 Swan Street, Buffalo. The chair ordered a ballot which resulted in the unanimous election of these candidates for membership.

Dr. Rochester said that the board of censors had done good work this year; among other things, securing convictions of noted criminals, and moved that the Medical Society of the County of Erie authorise the board to take such steps as may be necessary to annul the certificate of H. J. Ince now serving a term in the Erie County Penitentiary.

Dr. Grosvenor moved that nominations for all elective officers for the year 1909 shall be made at the June meeting of 1908. Carried.

A communication from the Medical Society of the County of Genesee, relative to the garnishee law, and also a resolution passed by said society in relation thereto was read by the secretary, and on motion was ordered referred to the committee on legislation with power.

A communication from Dr. Arthur McDonald, Washington, D.C., in relation to "A Plan for the Study of Man" was referred to the chairman of the committee on public health.

The society then adjourned.

